# A school-based program to prevent depressive symptoms and strengthen well-being among pre-vocational students (Happy Lessons): protocol for a cluster randomized controlled trial and implementation study

**DOI:** 10.1186/s12889-021-12321-3

**Published:** 2022-01-20

**Authors:** Marieke Rombouts, Elisa L. Duinhof, Marloes Kleinjan, Jannis T. Kraiss, Laura Shields-Zeeman, Karin Monshouwer

**Affiliations:** 1 Trimbos Institute, Netherlands Institute for Mental Health and Addiction, Utrecht, The Netherlands; 2 Department of Interdisciplinary Social Sciences, Utrecht University, Utrecht, The Netherlands; 3 Department of Psychology, Health, and Technology, Faculty of Behavioral, Management, and Social Sciences, University of Twente, Enschede, The Netherlands

**Keywords:** Depression prevention, Well-being promotion, Adolescents, Pre-vocational, School-based intervention, Integrated universal and indicated prevention

## Abstract

**Background:**

Depression is one of the leading causes of illness and disability among young people. In the Netherlands, one in twelve Dutch adolescents has experienced depression in the last 12 months. Pre-vocational students are at higher risk for elevated depressive symptoms. Effective interventions, especially for this risk group, are therefore needed to prevent the onset of depression or mitigate the adverse long-term effects of depression. The aim of this study is to examine the effectiveness and implementation of a school-based program Happy Lessons (HL), that aims to prevent depression and promote well-being among pre-vocational students**.**

**Methods:**

A cluster randomized controlled trial (RCT) with students randomized to HL or to care as usual will be conducted. Pre-vocational students in their first or second year (aged 12 to 14) will participate in the study. Subjects in both conditions will complete assessments at baseline and at 3- and 6-months follow-up. The primary outcome will be depressive symptoms using the Center for Epidemiologic Studies Depression Scale (CES-D) at 6-months follow-up. Secondary outcomes are well-being using the Warwick-Edinburgh Mental Well-Being Scale (WEMWBS) and life satisfaction (Cantril Ladder) measured at 6-months follow-up. Alongside the trial, an implementation study will be conducted to evaluate the implementation of HL, using both quantitative and qualitative methods (interviews, survey, and classroom observations).

**Discussion:**

The results from both the RCT and implementation study will contribute to the limited evidence base on effective school-based interventions for the prevention of depression and promotion of well-being among pre-vocational adolescents. In addition, insights from the implementation study will aid identifying factors relevant for optimizing the future implementation and scale-up of HL to other schools and contexts.

**Trial registration:**

This study was registered on 20 September 2021 in the Dutch Trial Register (NL9732).

**Supplementary Information:**

The online version contains supplementary material available at 10.1186/s12889-021-12321-3.

## Background

Worldwide, depression is one of the leading causes of illness and disability among young people [1]. In the Netherlands, one in twelve adolescents reported experiencing depression in the last 12 months [2]. Adolescent depression contributes to behavioral and educational difficulties such as school absenteeism, poor educational attainment, social problems, substance misuse, and suicidality [3–7]. Depression also has a considerable economic impact in terms of healthcare consumption [8, 9]. In 2017, the costs in the Netherlands that were associated with depression were 1.13 billion euros [10].

Studies show that approximately 50% of adults with a depressive disorder experienced their first depressive episode during childhood or adolescence [11]. The average age among Dutch adolescents for the first episode of depression is 14 years [2]. Mental illness among adolescents tend to persist into adulthood [12]. Accessible, evidence-based interventions aimed at the early detection of symptoms of depression and early intervention are thus crucial to help prevent depression and mitigate the negative long-term effects of depression [13].

In addition to early detection and intervention, improving adolescent well-being is important in mental health promotion [14]. Prior research showed that low levels of well-being are a risk factor for developing depression [15]. Thus, focusing on improving adolescent well-being in general may help reduce the risk of developing depression. Moreover, well-being was found to be associated with a healthier and longer life [16, 17], prosocial behavior, and good relationships with family and friends [16].

Yet, adolescents are typically not inclined to seek help for their mental problems. They have difficulties recognizing problem symptoms, want to solve problems themselves and fear being stigmatized [18, 19]. Providing interventions in social contexts, such as schools, can facilitate getting in touch with hard-to-reach groups, like adolescents. To date, effective school-based programs in the Netherlands that focus both on the prevention of depression and promotion of well-being are scarce and this is especially so for programs targeting the risk group of students in pre-vocational training, who go on to pursue more applied studies in post-secondary vocational education [20]. This is an omission as pre-vocational students are at higher risk for elevated depressive symptoms compared to adolescents in pre-university education [21]. Interventions aimed to improve mental health or well-being of high risk groups are not only scare in the Netherlands, but also internationally [22].

Therefore, effective well-being and depression prevention interventions that are tailored to the needs of these students are needed. This study tries to fill these gaps by examining the effectiveness and implementation of Happy Lessons (HL): a school-based program that focuses on both the prevention of depressive symptoms and the promotion of well-being among pre-vocational adolescents. HL offers an integral and stepped-care approach that combines universal and indicated prevention elements and is delivered by trained mental health professionals within the school context. HL is designed to promote well-being and to identify and detect students that are at risk of developing depression or experiencing depression and facilitate referral to appropriate local mental health and social care services.

The goal of this paper is to describe the protocol for a study that will test the effectiveness of the HL-program in a Dutch adolescent sample of pre-vocational students through a cluster randomized controlled trial (RCT). In addition, an implementation study will be carried out to evaluate the implementation of HL and identify factors that may have influenced implementation.

## Methods

This protocol was developed in accordance with the Standard Protocol Items: Recommendations for Interventional Trials (SPIRIT) statement [23]. See Appendix 1 for the SPIRIT checklist.

### Aim

The main objective of the RCT is to investigate the effectiveness of HL in terms of a reduction of depressive symptoms in pre-vocational students aged 12 to 14 after 6 months compared to care as usual. We hypothesize that adolescents who participate in the HL-program will have lower levels of depressive symptoms at 6-months follow-up than adolescents who do not participate. Secondary outcomes that will be investigated are well-being and life satisfaction. We hypothesize that students who receive HL report higher levels of well-being and life satisfaction than students who do not receive HL.

In addition, we want to identify potential mediator factors that increase or decrease the intervention’s effectiveness. We will investigate school and class climate indicators. We hypothesize that students who receive HL will report higher levels of classmate support, teacher support, and school connectedness, and lower levels of bullying, which will lead to a reduction of depressive symptoms among students.

A secondary aim of this study is to evaluate the implementation process of the HL-program, from the perspectives of intervention recipients, implementers, and the broader stakeholders at the school level (e.g. teachers). The aim of this evaluation will be to understand how the intervention was implemented and what factors might be important when sustaining or expanding the HL-program over time or in new schools.

### Study setting

In the Netherlands, pre-vocational education is a four-year program offering theoretical and practical courses (in Dutch: VMBO). This study will be conducted among first- and second-grade pre-vocational Dutch students. Students in these classes are between 12 and 14 years of age.

### Happy Lessons

#### Theoretical basis

HL is a school-based program designed to prevent depressive symptoms and promote well-being among young people. It is specifically developed for (pre-)vocational students. Prior school-based depression prevention programs have been associated with reduction in depressive symptoms, although effects are typically small [24, 25].

HL contains a unique combination of key elements that can increase program-effects. First, prior research showed that interventions that work well in schools are programs that are shorter in duration, targeted towards high-risk students, and delivered by professionals interventionists instead of classroom teachers [24, 25]. HL is a compact program, focuses on detection elevated depression levels among youth, and is delivered by trained mental health professionals instead of teachers.

Second, mental health prevention programs that combine universal and indicated elements show larger effect sizes [25, 26]. HL meets this criterion as well: it is an integrated approach that combines universal and indicated elements. HL consists of four classical lessons (including two e-learnings), an online screener (called the HL-questionnaire) and an individual advice session for all students in which the at-risk group will be referred to local indicated prevention or treatment.

Third, research shows that ‘positive framing and goal setting’ are important to reduce fear of stigma and encourage active problem solving [27] and that programs with a positive mental health focus are more effective [26]. HL is based on positive psychology and cognitive behavioral therapy, both effective methods in the treatment of depressive symptoms [28–30].

Fourth, for successful implementation, it is important that a program is embedded in the workflow of the stakeholders. Effective implementation is associated with larger effect sizes [26]. The HL-pilots show the program fits in the workflow of the mental health professionals who provide HL. The lessons and advice sessions can be delivered in ‘mentor hours’ at the school, and the HL advice session may be combined with the periodic health screens or ‘adolescent contact moments’ that are conducted by the youth health authorities in the Netherlands. And because of its compactness, HL may be integrated in a bigger lifestyle programs in schools with other lifestyle themes.

Finally, HL is also relevant for the mentally healthy students in the classroom who do not need to improve on depression and well-being outcomes. Should they experience mental health problems in the future, they will have received the message with HL that mental health problems are a normal part of life, that they don't have to feel ashamed, that there are effective skills that can be practiced and that good help is available [31].

#### Program

**Fig. 1:**
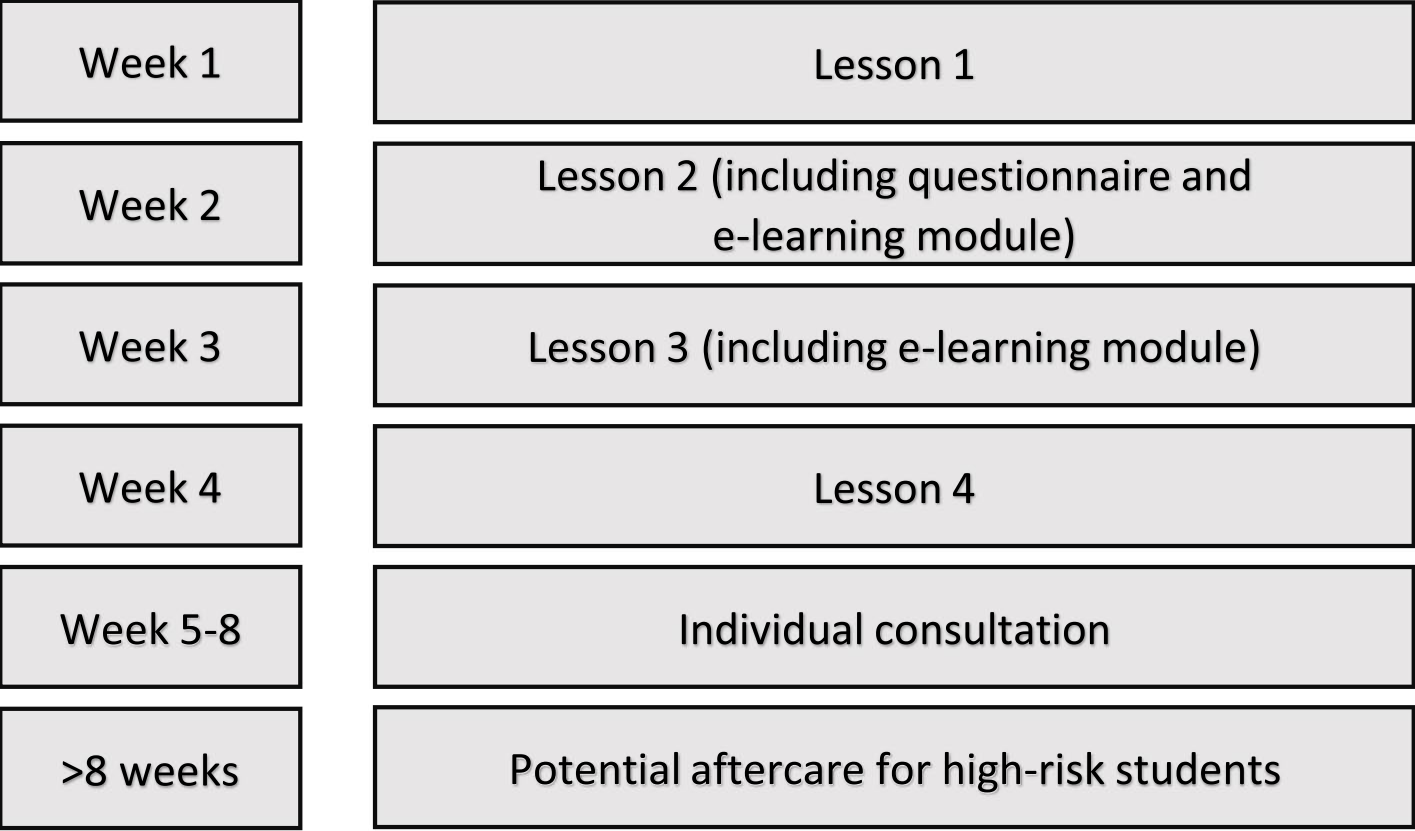
Components of the HL-program

The program consists of various parts (see Fig. 1): (a) four classroom lessons of which two lessons also contain an e-learning module, (b) an online HL-questionnaire that includes two well-being scales and a depression scale that is administered at the start of the second lesson, (c) an individual consultation session with the mental health professional who delivers HL for each student, and (d) potential aftercare for high-risk students. The program is delivered within approximately 5-8 weeks. The lessons and advice sessions are provided by trained mental health professionals (e.g., prevention workers, called HL-trainers). Teachers are present to support the HL-trainers in providing HL. Before the start of the program, HL-trainers create a personal HL-account for each student in an online database. Students will use the HL-account to gain access to the online HL-questionnaire and e-learning module (lesson 2 and 3).

##### The classroom lessons

The first lesson focuses on the introduction of HL and on the subject happiness. The goal of this first lesson is to encourage a discussion about feeling happy or unhappy, familiarize students with different aspects of happiness, discuss the difference between happiness and being lucky, and to break the taboo about feeling unhappy or down. In this context, engaging group assignments and discussions take place.

In the second lesson, students will learn more about the things that might make them happy and the relationship between feelings, thoughts, and actions. In addition to a class-based exercise, students are asked to complete the online HL-questionnaire. This is a questionnaire on demographics (i.e., gender, age), their own health, family situation (i.e., their family’s’ financial situation, and their family members’ (mental) health status), a depression scale, life satisfaction scale, and a well-being scale. After students complete the HL-questionnaire, they will start the e-learning module which contains videos, animations, and fun and engaging exercises. The e-learning module helps students to improve their mood and to practice the skills with their own feelings, thoughts, and actions.

The third lesson starts with an exercise where the HL-trainer describes different situations (e.g., when students receive the results from a test or students have to give a presentation). Students are asked to describe their feelings for each situation (i.e., being angry, sad, scared, or happy). The lesson also contains an e-learning module with the same elements as the second lesson but is focused on: (1) problem solving, (2) communication tips that help students maintain positive relationships with friends, parents, teachers, (3) the importance of helping others, and (4) thinking about their future dreams and how they can achieve these.

The fourth and final lesson includes a recap of what students have learned so far. In addition, it focusses on the relationship between thoughts and feelings and how students can challenge negative thoughts and change them into more helpful thoughts. Students are also asked to fill in a short evaluation questionnaire about the program. The duration of each lesson is approximately 45 minutes.

##### The individual consultation session and potential aftercare

After the final lesson, individual consultation sessions are scheduled with all students. In the online database, the HL-trainer can see the answers of the HL-questionnaire. Based on the answers on the HL-questionnaire, the HL-trainer talks with the student to see if the student needs additional support. Students who report high scores on the HL-questionnaire or report other problems at home/school are offered help by the HL-trainer (i.e., some additional individual sessions) or are referred to appropriate local mental health and/or social care services. This will be done in consultation with the student and their parent(s).

### Design of the RCT

The effectiveness of HL will be tested within a cluster RCT with two conditions: an intervention condition (receiving HL during the study) and a control condition (care as usual, HL will be delivered after the final follow-up assessment). Participating classes will be randomly assigned to either the intervention or the control condition and will be equally distributed across the two conditions within each schoolyear. This design is chosen to avoid a skewed distribution of participants over the two conditions due to school effects. The randomization will be conducted by an independent statistician. Three assessments will be performed in the study: T0 (pre-test, before the start of HL in intervention group); T1 (3-months follow up); T2 (6-months follow-up).

Data will be collected in two cohorts: the first cohort is planned in the 2021-2022 school year and the second cohort is planned in 2022-2023 school year.

**Fig. 2:**
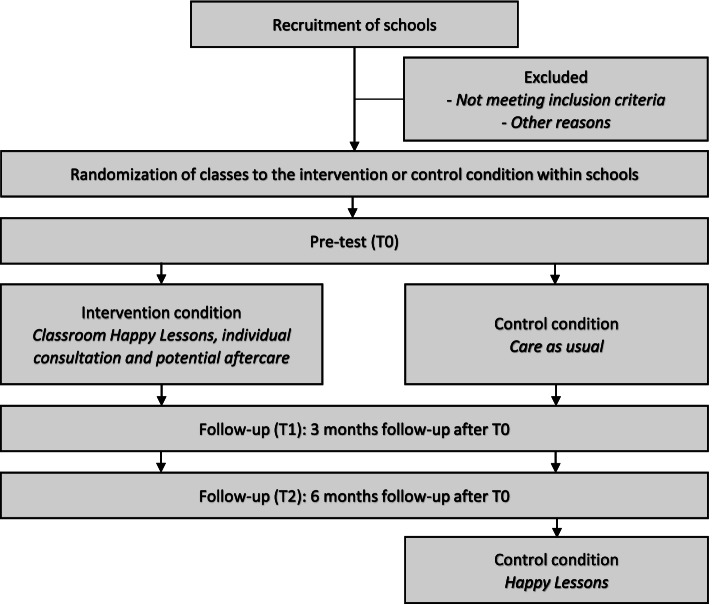
Flowchart of the study design

See Fig. 2 for the flowchart of the study and Table 1 for an overview of the schedule of enrolment, interventions, and assessment.

**Table 1:**
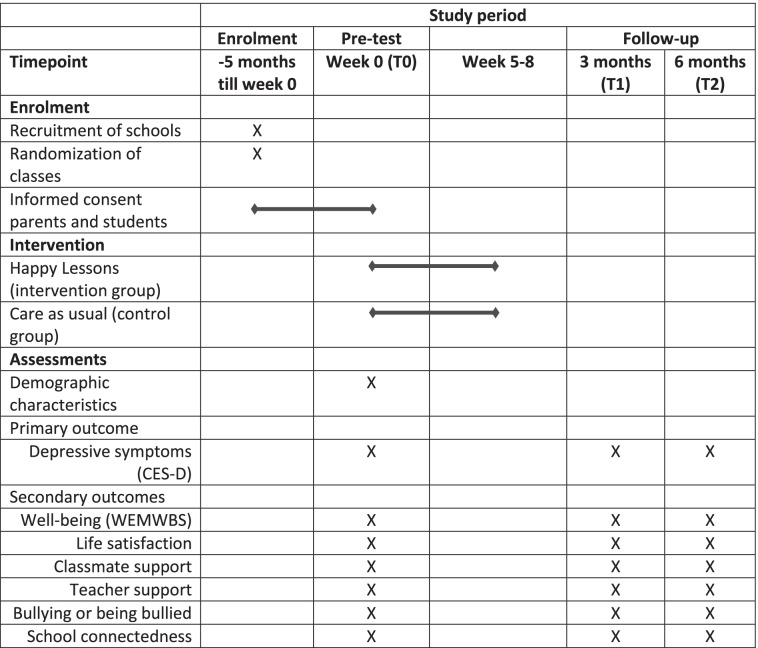
Schedule of enrolment, intervention, and assessments

#### Study population

##### Eligibility criteria and recruitment of schools

To provide HL at a school, three different parties need to be involved: schools, mental health organizations that provide the professionals that deliver HL, and – in most cases – the municipality that pays for HL. Schools, mental health organizations, and municipalities were recruited via the website and newsletter of the Trimbos Institute and the HL website. Schools are included in the study if they offer pre-vocational education in at least two first-year or two second-year classes (age 12-14 years). This may also include “combination classes” that contain students from both pre-vocational and higher educational levels.

##### Eligibility criteria of participants

Students are eligible to participate in the study when they meet the following inclusion criteria: enrolment of first- or second-grade students of classes that provide pre-vocational education (including combination classes), informed consent from student and parent(s) and a sufficient knowledge of the Dutch language.

#### Recruitment of participants and informed consent

Two weeks before the start of the study, schools provide information letters and consent forms to parents from both groups (intervention and control condition). In order to give permission for their child’s participation, parents are asked to sign the consent form on paper and send it back to the research team by mail or e-mail within a week. Approximately one week before the start of HL in the intervention condition, a research assistant will visit the participating classes to explain the study and provide an information letter to all students. Students can ask questions and are asked to sign an informed consent form on paper in this recruitment session. Students will be enrolled in HL when they and their parents have both provided consent. In consultation with the school, participating students will receive an incentive (e.g., a voucher).

#### Sample size

The sample size is based on the main outcome: depressive symptoms in pre-vocational students in first and second year classes. Based on a meta-analysis by Stice et al. [24], we assume an effect size of *d* = 0.20. Thus, we want to test the hypothesis that we will find an effect of *d *= 0.20 (or larger) at 6 months follow-up. This will be tested at a two-sided significance level of 5% and a power of 80%. Accounting for the clustering in the data with 22 students on average per class, an intraclass correlation of 0.02, a cluster size variation of 0.17 and a baseline correlation of 0.30, we need *n* = 315 students in each condition. Furthermore, we expect that approximately 40% of parents and/or students will not provide informed consent. In order to secure the needed sample size, the aim is to sample 525 students per trial. Assuming an average of 4 classes of 22 students per school (divided across intervention and control condition), this will require 48 classes from about 12 schools.

The sample size calculation was performed in Stata version 14.2 statistical software package using the clustersampsi-procedure. The intraclass correlation coefficient (ICC) of 0.02 was based on two studies: the Dutch Health Behaviour in School-aged Children Survey (HBSC) [32], where the ICC on the emotional scale within the Strengths and Difficulties Questionnaire (SDQ) was 0.008 (95% CI: 0.003-0.02) and a large European study where the ICC’s for depressive mood varied across countries from 0.01 to 0.07 [33].

#### Data collection

Data will be collected through an online questionnaire and students’ self-reports. Students will use their personal HL-accounts (created by the HL-trainer) to gain access to the questionnaires. Data for each assessment and in both conditions will be collected during a regular classroom lesson. At the end of the recruitment session, the research assistant will ask all students to fill in the HL-questionnaire as part of the HL-program. For those participating in the study this data will serve as the baseline measurement (T0) for the RCT. For research purposes, the HL-questionnaire is administered before the start of the HL instead of the second lesson. For the second (T1) and third assessment (T2), teachers are approached to collect and administer the questionnaires in their classes. The duration of each questionnaire will be approximately 15 minutes.

Data collected for research purposes will be stored in a database. For each individual student, a unique respondent number will be used. Students’ names and email addresses will be stored in a separate database, which is only accessible for the data manager of the project.

#### Measures

##### Demographic characteristics

*Demographic characteristics* include gender, birth month and -year and immigration background. Immigration background is assessed by asking the students to indicate their own and both of their parents’ country of birth. Students are considered to have an immigration background if they or at least one of the parents was born in another country then the Netherlands. Students’ educational level is collected via the school.

##### Primary outcome

*Depressive symptoms* will be measured with the Dutch version of the Center for Epidemiologic Studies Depression Scale (CES-D) [34, 35]. The CES-D measures the frequency of 20 depressive symptoms over the past week on a four-point Likert scale. Examples of items are: “I enjoyed life” and “I felt that everything I did was an effort”. Answer categories are: (0) “rarely or none of the time (less than once a week)”, (1) “some or a little of the time (1-2 days a week)”, (2) “occasionally or a moderate amount of time (3-4 days a week)”, or (3) “most or all of the time (5-7 days a week)”. The total score may range from 0 to 60, with higher scores indicating higher levels of depression. The Dutch version has been validated among adolescents and has excellent psychometric properties [31, 36]. Based on previous Dutch findings, we will use a CES-D cut-off score of 16 or higher to identify students with subclinical levels [34] and a cut-off score of 22 or higher to identify students with clinical levels of depression [36].

##### Secondary outcomes

*Well-being* will be measured with the Dutch translation of the Warwick-Edinburgh Mental Well-Being Scale (WEMWBS) [37, 38]. The WEMWBS assesses 14 items on a five-point scale. Scores range from 14 to 70, with higher values reflecting greater perceived well-being. Questions concern feelings experienced in the previous two weeks, such as “I felt good about myself” and “I felt optimistic about the future”. Studies from the United Kingdom found that it is appropriate to use the 14-item WEMWBS to measure well-being among adolescents aged 13 to 16 [39]. In two earlier pilot studies of HL involving pre-vocational students aged 13 to 17, the WEMWBS showed high reliability with Cronbach’s Alpha of 0.84 and higher [31]. The WEMWBS can detect meaningful change at both the group and individual level. At the individual level, minimum detectable change has been set at a change of 3 points [40].

*Life satisfaction* will be assessed with the Cantril Ladder [41]. Students rate how satisfied they are with their life on a visual analogous scale ranging from (0) “the worst possible life” to [10] “the best possible life”. The Cantril Ladder is easily understood and has shown good reliability and convergent validity among young people (e.g., [42]).

*Classmate support* will be assessed by three items indicating the extent to which classmates were experienced as supportive, e.g., “Most of the students in my class are kind and helpful” (Response categories ranging from (1) “completely agree” to (5) “completely disagree”). This is a cross-nationally valid and reliable scale for young people [43].

*Teacher support* will be assessed by three items indicating the extent to which teachers were experienced as supportive, e.g., “I feel that teachers accept me as I am” (Response categories ranging from (1) “completely agree” to (5) “completely disagree”). Cross-national studies showed the scale to be valid and reliable for young people (e.g., [42]).

*Bullying involvement*. Students are asked how many times they were involved in bullying at school in the past four weeks. The response categories available are: (1) “zero times”, (2) “ones or twice”, (3) “about once a week”, (4) “several times a week”. This is a validated measure on bullying involvement that shows good psychometric properties [44].

*Bullying victimization.* Students are asked how many times they have been bullied at school in the past four weeks. Response categories include: (1) “zero times”, (2) “ones or twice”, (3) “about once a week”, (4) “several times a week”. This is one of the most validated measures on bullying victimization [45] with sound construct validity [44].

*School connectedness.* The Psychological Sense of School Membership Scale (PSSM) [46] assesses students’ connectedness with their school. It has 18 items, and a Dutch translation is available. A shortened, 2-item version has been administered in one Dutch study, showing a satisfactory internal consistency of .76 [47]. This short version contains the items: “I feel like a real part of my school” and “I feel proud of belonging to my school”. In this study, this short version will be used.

#### Statistical analyses

All statistical analyses will be conducted in R [48] by an independent researcher. Reporting of the results will be conducted in accordance with the (extended) CONSORT statement [23]. Analyses will be conducted based on the intention-to-treat (ITT) principle. The primary ITT analyses will be performed using linear mixed models, which adequately deal with missing at random data and can be used to account for the nested structure of the data; students nested within classes and repeated assessments nested within students [49]. Time, condition, and time by condition interactions will be specified as fixed effects to examine the effectiveness of the intervention on continuous primary and secondary outcomes. Baseline scores of the corresponding outcome will simultaneously be entered as fixed factors in the model to account for baseline imbalances. In accordance with the CONSORT statement [23], it will not be explicitly tested whether conditions statistically differ in their baseline characteristics. Hence, the decision whether models will be adjusted for additional covariates will not be based on whether statistical differences in these covariates exist at baseline. Instead, models will primarily be reported that are not adjusted for additional covariates. In an additional sensitivity analysis, it will be examined whether adjusting these models for the following baseline variables substantially changes conclusions drawn from the analyses: gender, age, immigration background, depressive symptoms, well-being, and life satisfaction. Post-hoc tests will be conducted to examine between-group differences at each timepoint using analyses of covariance (ANCOVA), including baseline scores as covariate. Based on estimated marginal means and corresponding standard errors from linear mixed models, between-group effect sizes at each timepoint will additionally be expressed as Cohen’s *d* with 95% confidence intervals.

Mediation analyses will be performed to examine potential working mechanisms of the effect of the intervention. Research has shown that there is an association between school climate and mental health of students [50]. Positive relationships between students and their teachers or peers increased psychosocial well-being [50]. Moreover, teacher support reduced depressive symptoms [51]. In addition, higher school connectedness (attachment and belonging to school) was positively associated with students’ psychosocial well-being [50] and acted as a buffer against depressive symptoms and anxiety [51, 52]. Finally, causal associations were found between bullying and depression [53]. Therefore, the following variables will be tested as potential mediators: classmate support, teacher support, school connectedness, and bullying. The categorical variable condition will be included as independent variable in all mediation models (0 = control, 1 = intervention), and the observed scores of primary or secondary outcomes at follow-up as dependent variable. Total, direct, and (total) indirect effects with 95% bootstrapped confidence intervals will be calculated for all mediation models [54, 55].

Furthermore, exploratory moderator analyses will be conducted to examine whether the effect of the intervention on primary and secondary outcomes depends on a priori defined variables. Various sociodemographic factors have an influence on interventions focused on depressive symptoms or well-being. Research has shown that girls might experience more benefits from depression prevention programs than boys [56]. Moreover, older participants might benefit more from cognitive-behavioral therapy [24, 57]. In addition, research has shown that depression prevention programs are likely to be more effective for adolescents with ethnic minority backgrounds [24], since they report more problems with mental health compared to adolescents with ethnic majority backgrounds [32]. Therefore, the following variables will be examined as potential moderators: level of baseline CES-D depressive symptoms, gender, age, and immigration background. Separate linear mixed models with condition, the putative moderator, and the interaction of these two as fixed effects will be used to examine potential moderation effects.

### Design of the implementation study

#### Study population

The implementation study will be executed among recipients of HL (students), implementers (HL-trainers), and other school professionals (e.g., teachers, care coordinators).

#### Measures

As the aim is to understand the implementation process and how the intervention was implemented and received, we relate to implementation constructs from Proctor’s Implementation Outcome Framework [58], focusing on fidelity, acceptability, appropriateness and feasibility. The implementation process measures, and their data collection methods are shown in Table 2.

**Table 2:** Operationalized implementation measures and data sources

Implementation measure	Operationalization of measure	Data sources
Fidelity	Extent to which the program was delivered as planned/trained by HL-trainers.	Classroom observations Interviews with HL-trainers
Appropriateness	The relevance, suitability, and usefulness of the program. This includes the fit of the program to the classroom context, perceived utility from among recipients and implementers.	Students’ evaluation survey Interviews with HL-trainers
Acceptability	Degree of satisfaction of students, schools, and HL-trainers with the program.	Students’ evaluation survey Interviews with HL-trainers Interviews with school professionals
Feasibility	Extent to which HL-trainers and school professionals perceive the program to be feasible to deliver in other contexts, as well as fit of the program to the classroom setting.	Interviews with HL-trainers Interviews with school professionals

#### Data collection and analysis

As shown in Table 2, multiple sources of data collection will be used. Data will be collected through classroom observations (*n* = 20), qualitative interviews with HL-trainers (*n* = 10) and teachers or care coordinators (*n* = 10), or until saturation is reached, and an evaluation survey for students.

Classroom observations are conducted to investigate to which extent the program was delivered as planned/trained by HL-trainers. An observation checklist will be made based on components of the instruction manual for HL-trainers (e.g., if the exercises were conducted and executed within the allocated time). During the observation, additional notes will be made (for example, on HL-trainer and student interaction, classroom engagement). For each lesson, a different checklist is designed, tailored to the different exercises and goals. We will conduct observations in five classes and within each class, we will observe all four lessons. For the classroom observations, checklist items, and items with scores or response items will be analysed quantitatively through descriptive statistics and frequency tables. For the open-ended items of the observations, we will perform a qualitative analysis, looking at common themes across answers.

Semi-structured interviews will be carried out with HL-trainers and school professionals to understand their experience with receiving or delivering the program, including how feasible the content was to deliver, how satisfied students were with the content of the program, adaptations made to the program by HL-trainers, etc. Interviews will be audio-taped with participants’ permission and transcribed verbatim. Qualitative analysis will be carried out by two independent researchers according to a coding framework developed by the project team. Coding will take both a deductive and inductive approach and a thematic content analysis approach will be applied to coded data.

Students will be asked about their experience with the HL-program through an online survey. Survey items will include closed and open-ended questions with open fields for answers. Survey items will include what they have learned from the program, if they would recommend HL to peers, if they were satisfied with the program and suggestions for improvement. The closed questions will be analysed in SPSS 27.0. Descriptive statistics will be reported. For the open-ended questions, an inductive approach will be used.

## Discussion

This protocol paper outlines a cluster RCT and an implementation study that together aim to assess the effectiveness and implementation of a school-based program for pre-vocational adolescents in the Netherlands designed to prevent depression and promote well-being. The primary aim is to investigate levels of depressive symptoms at 6-months follow up among students who receive HL compared to students who received care as usual. Secondary aims include assessing the effects of HL on well-being and life satisfaction at 6-months follow up, identifying whether school and class climate mediate the effect of HL on depression, exploring potential effect-modifying factors (i.e., demographic factors and symptoms at baseline), and evaluating the implementation of the intervention for improvements and possible scale-up.

### Strengths and limitations

A strength of this study is that we will perform the study in a real-life condition, which will increase the external validity of the results. Furthermore, we will not only focus on the effectiveness of the program but will also study potential mediators and moderators. This will give us insight into why and for whom the program is effective. Another strength is that we also look at implementation outcomes, to be able to distil key factors affecting implementation for other schools considering implementation of HL.

Despite these strengths, this study does not come without limitations. Since we randomize at the classroom level, a possible negative effect could be that control students are ‘contaminated’ from contact with and possible influence from students or school mates in the intervention arm. We expect however that these effects are minimal and will have no meaningful effect on the outcomes of this study. Another limitation is that we did not use a representative sample of schools to investigate the effectiveness of this program. We expect that schools which more interested in promoting well-being and preventing depressive symptoms among their students, are more likely to participate in this program and study. In addition, to get an impression of the representativeness of our sample, we will compare the results of this study (e.g. life satisfaction) with data from national studies [59, 60]. Furthermore, we decided to include a 6-month follow-up measurement, and not a 12-months measurement since it was preferred by schools to logistically complete the program and its evaluation within one school year. Another limitation of the study is that the students’ results are based on self-reported data, which might lead to measurement errors or under-reporting of depressive symptoms due to social desirability bias. However, we will use validated questionnaires or questionnaires used in other studies.

### Implications for practice

The results from both the RCT and implementation study will contribute to the evidence base on school-based interventions for depression and promoting well-being among pre-vocational students, which is scarce. In addition, the results will be useful for optimizing future implementation of the program and elucidate insights for further scale-up to other schools and contexts. With the results of the implementation study, we will be able to understand the factors that will affect implementation, which can be used to better tailor and target an implementation plan for HL-trainers and schools in the future. This information will also be of interest for other similar school-based interventions.

## Supplementary Information

**Additional file 1. **SPIRIT 2013 Checklist: Recommended items to address in a clinical trial protocol and related documents.

## Data Availability

Not applicable.

## Abbreviations

CES-D: Center for Epidemiologic Studies Depression Scale
HL: Happy Lessons
ICC: Intraclass correlation coefficient
RCT: Randomized controlled trial
SPIRIT: Standard Protocol Items, Recommendations for Interventional Trials
T0: Pre-test
T1: 3-months follow-up
T2: 6-months follow-up
WEMWBS: Warwick-Edinburgh Mental Well-Being Scale
